# HISTORICAL CHANGE IN MIDLIFE TRAJECTORIES OF PHYSICAL HEALTH: A CROSS-NATIONAL APPROACH

**DOI:** 10.1093/geroni/igae098.1782

**Published:** 2024-12-31

**Authors:** Yesenia Cruz-Carrillo, Frank Infurna, Nutifafa Dey, Markus Wettstein, Kevin Grimm, Margie Lachman, Denis Gerstorf

**Affiliations:** Arizona State University, Tempe, Arizona, United States; Arizona State University, Tempe, Arizona, United States; Arizona State University, Tempe, Arizona, United States; Humboldt University Berlin, Berlin, Berlin, Germany; Arizona State University, Tempe, Arizona, United States; Brandeis University, Waltham, Massachusetts, United States; Humboldt University Berlin, Berlin, Berlin, Germany

## Abstract

Empirical evidence has documented that later–born cohorts of middle–aged adults in the U.S. are reporting poorer mental and physical health than earlier–born cohorts. However, less is known about whether this is specific to the U.S. or generalizes to other nations and to what extent this is transpiring across self–report and objective indicators of physical health. Our study aims to describe the similarities and differences in historical change of midlife development in self–rated health, health conditions, and grip strength by using harmonized data from longitudinal panel surveys from the U.S. (HRS), Mexico (MHAS), 13 nations in Europe (ELSA, SHARE), South Korea (KLoSA), and China (CHARLS). Results from growth curve models revealed historical declines in self–rated health and grip strength (for both men and women), and historical increases in health conditions among middle–aged adults from the U.S. In contrast, historical improvements in self–rated health and fewer health conditions were observed for middle–aged adults in Mexico, Europe, South Korea, and China. Results for grip strength are more nuanced, with historical improvements among later–born cohorts of both men and women in Nordic Europe and South Korea and historical declines among both men and women in China. Our discussion focuses on better understanding these national differences in historical trends by considering how the dynamically changing historical context impacts ways of living and health care systems as well as considering the often-profound implications arising for psychological functioning and development at the individual and population level.

